# Treatment Approaches for Functional Neurological Disorders in Children

**DOI:** 10.1007/s11940-022-00708-5

**Published:** 2022-03-28

**Authors:** Areti Vassilopoulos, Shekeeb Mohammad, Leon Dure, Kasia Kozlowska, Aaron D. Fobian

**Affiliations:** 1 Yale Child Study Center at Yale School of Medicine, New Haven, CT USA; 2 TY Nelson Department of Neurology and Neurosurgery, The Children’s Hospital at Westmead, Sydney, NSW 2145 Australia; 3 Children’s Hospital at Westmead Clinical School at Sydney Medical School, Faculty of Medicine and Health, University of Sydney, NSW 2145 Westmead, Australia; 4 Departments of Pediatrics and Neurology, University of Alabama at Birmingham, Birmingham, AL USA; 5 Department of Psychological Medicine, The Children’s Hospital at Westmead, Sydney, NSW 2145 Australia; 6 Discipline of Psychiatry and of Child & Adolescent Health, Westmead Institute for Medical Research, Sydney Medical School, Westmead, NSW 2145 Australia; 7 Department of Psychiatry, University of Alabama at Birmingham, Birmingham, AL USA

**Keywords:** Functional neurological disorder (FND), Treatment, Pediatric, Functional seizures, Psychogenic non-epileptic seizures, Clinical ethics

## Abstract

**Purpose of Review:**

Functional neurological disorder (FND) is a multi-network brain disorder that encompasses a broad range of neurological symptoms. FND is common in pediatric practice. It places substantial strains on children, families, and health care systems. Treatment begins at assessment, which requires the following: the *medical* task of making the diagnosis, the *interpersonal* task of engaging the child and family so that they feel heard and respected, the *communication* task of communicating and explaining the diagnosis, and the *logistical* task of organizing treatment.

**Recent Findings:**

Over the past decade, three treatment approaches—Retraining and Control Therapy (ReACT), other cognitive-behavioral therapies, and multidisciplinary rehabilitation—have been evaluated in the USA, Canada, and Australia. Of children treated in such programs, 63 − 95% showed full resolution of FND symptoms. The common thread across the programs is their biopsychosocial approach—consideration of biological, psychological, relational, and school-related factors that contribute to the child’s clinical presentation.

**Summary:**

Current research strongly supports a biopsychosocial approach to pediatric FND and provides a foundation for a stepped approach to treatment. Stepped care is initially tailored to the needs of the individual child (and family) based on the pattern and severity of FND presentation. The level of care and type of intervention may then be adjusted to consider the child’s response, over time, to treatment or treatment combinations. Future research is needed to confirm effective treatment targets, to inform the development of stepped care, and to improve methodologies that can assess the efficacy of stepped-care interventions.

## Introduction

Functional neurological disorder (FND) is a multi-network brain disorder that encompasses a broad range of neurological symptoms [1–3]. Presentations with FND are common in pediatric practice—up to 10% of children presenting to pediatric neurology clinics [4] and up to 20% of children presenting to specialist epilepsy clinics [5]. FND places substantial psychosocial, educational, and financial strains on children and their families and a substantial burden on the health care system [6, 7].

Motor FND and functional seizures (FS)^1^ are the two most common patterns of FND presentations in children [8, 9]. Motor FND in children, sometimes comorbid with FS or sensory symptoms, affects the function of the skeletal muscles—muscles that are normally under voluntary control. These presentations include functional limb weakness/paresis, functional movement disorders (uncoordinated or bizarre gaits, functional tremor, tics, chorea, myoclonus, dystonia, and abnormal movements affecting the eyes, face, and jaw), functional voice disorders, swallowing difficulties, regurgitation, and cough. Interestingly, the number of functional tic presentations has surged in 2020 − 2021 in the context of the COVID-19 pandemic [10, 11], highlighting the biopsychosocial nature of the disorder and the complex interactions between brain, mind, and body and context. FS take a wide variety of forms—for example, episodic unresponsiveness, shaking of limbs, loss of muscle tone, faint-like events, and altered awareness.

The diagnostic process for FND is often undertaken by practitioners (typically neurologists) using current diagnostic criteria [12, 13]. The diagnosis is a positive diagnosis: the neurologist elicits, and relies on, positive (rule-in) clinical signs to support the diagnosis (see next section). The ability of neurologists to accurately diagnose FND has been shown to be quite good, with one study finding that pediatric neurology residents providing consultations to the emergency room had approximately 94% accuracy in diagnosing pediatric FND [14].

While neurologists and other physicians play an important role early on, the treatment of pediatric FND is typically the purview of mental health and allied health professionals, including physical, occupational, speech, art, and recreational therapists. Until recently, research in pediatric FND was sparse, but the evidence base is now growing rapidly. This review aims to highlight some of that progress. After discussing the neurology assessment—with a particular focus on motor FND and FS—we review current treatment approaches and their efficacy and make suggestions for future research and the growth and development of treatment services.

## Approaches to the Diagnosis of FND: The Neurology Assessment

The treatment of pediatric FND begins with a neurology assessment conducted by a neurologist (or pediatrician) with the following four goals in mind [15, 16, 17]: the *medical* task of making the diagnosis (neurology examination and medical workup); the *interpersonal* task of engaging with the child and family so that they feel heard and respected; the *communication* task of explaining the findings of the assessment, communicating the diagnosis, and providing information about the treatment that the child needs in order to return to health and well-being; and the *logistical* task of organizing referrals to ensure that treatment is promptly implemented.

### Motor Functional Neurological Disorders

The neurology examination begins informally—in the waiting room, the corridor, or the examination room—with the neurologist taking note of the child’s motor function when the child is not being formally examined.

History taking is an important part of the neurology assessment. A preliminary question for the neurologist is whether symptom onset was sudden or insidious. If onset was sudden, then the neurologist is likely to include motor FND high on the differential diagnosis. The neurologist will also ask about the degree of disability and the impact on the child’s day-to-day functioning.

After the history is complete, the neurologist begins the examination proper, where they observe or elicit positive (rule-in) signs characteristic of motor FND, enabling the neurologist to make a positive diagnosis. Positive signs and examination techniques that support a motor FND diagnosis include the following:

- Discrepancies between the child’s movements or level of disability when the child’s attention is directed to the symptoms and when they are engaging in automatic tasks such as when checking text messages or during pauses in the examination
- Distribution of weakness that is not congruent with a neurological pathway (e.g., arm and leg weakness on opposite sides of the body)
- Entrainment of jerky movements (functional tremor, chorea, or myoclonus) with rhythmic movements of another body part
- Complete suppression of the functional movements on distraction or on contralateral ballismic movements requested by the examiner
- An unstable gait, when not a true ataxia (this can be distinguished from ataxia when the child walks with a narrow base or displays relatively fast and stable posture and movements when turning around or when bending over to pick up objects, while demonstrating extreme swaying or forced steps at other times)

For more information about the technical aspects of the neurology examination, see Espay and colleagues [18] and Kozlowska and colleagues [17].

It may be useful for the neurologist to videotape the examination (with the family’s permission) or ask for home video recordings of motor events. The recordings can then be reviewed with the child and family to highlight the positive (rule-in) signs that were elicited or observed and that support a diagnosis of motor FND. In presentations with functional tics—as seen more frequently during the current pandemic—teenage onset, female sex, lack of a premonitory urge, and comorbid anxiety, depression, or self-harm behaviors are other suggestive features [10, 11, 19].

In many contemporary settings, the neurology assessment includes a blood panel and may also include imaging. A baseline blood screen that includes iron studies, B12, vitamin D, thyroid function, and inflammatory markers (documenting low-grade inflammation) is important. Approximately two-thirds of children present with comorbid anxiety, depression, pain, fatigue, and other nonspecific somatic symptoms; factors that contribute to these conditions (e.g., iron deficiency in adolescent girls) may need to be addressed. While imaging may not be medically required, it can be helpful in allaying the concerns of the child, family, and treating team. Even if the results of any investigations are still pending at the time of the assessment, it is important for the neurologist to communicate the clinical diagnosis of motor FND and to discuss with the child and family that findings from the investigations are unlikely to change the diagnosis. Although ancillary investigations such as tremor studies and other neurophysiological tests are used in adult practice (for tremor and myoclonus), their applicability in children is limited by the need for sedation for electrophysiology and also by the lack of testing access and trained providers.

The prognosis of motor FND children is generally good (see studies reviewed in this article). One problematic area involves presentations with fixed dystonia, which is more difficult to treat, and with uncertain outcomes. In two studies with children and adults, 23% and 56.6%, respectively, had improved symptoms [20, 21]. In one child cohort—in which all had received intensive multidisciplinary treatment—85% had resolved [22].

#### Functional Seizures

As in motor FND, history taking is the first step of the neurology assessment and may involve viewing family-provided home videos of FS. Neurologists, especially those familiar with FS, are skilled at identifying clinical features suggestive of FS and, from the outset, include FS high on the differential diagnosis. Common features include asynchronous limb movements (various limbs moving at various times), long duration (e.g., > 10 min), ictal crying, and sudden resolution with no postictal alterations (e.g., confusion and disorientation) [18, 23–25]. If the child experiences an event during the consultation—or later during electroencephalogram (EEG)—the forced eyelid closure test (i.e., closed eyelids resisting passive opening), self-protective response to a threat stimulus (e.g., dropping the child’s hands over his/her face), or presence of preserved consciousness (during the event the child can hear what those around him or her are saying) [26] are other common features. Another clue suggestive of FS is events that occur only in certain places or situations (e.g., school).

The gold-standard assessment for FS includes a video EEG confirmation that the event is not associated with epileptiform changes [24]. In many clinical settings, access to video EEG may not be available. If so, the neurologist can still make a presumptive diagnosis based on history, clinical features, and an ordinary EEG.

Novel assessment techniques that do not require capturing an event on EEG are also being evaluated. One study found that children with FS (vs. children with epilepsy and healthy controls) maintain activation in the high-frequency bands of the EEG following 3 min of hyperventilation (a physiological stressor) [27]. These findings build on an earlier study finding that children with FS showed dysregulation of their respiratory motor systems and that half the children in the study triggered their FS by hyperventilation [28]. An adult study has recently reported that a panel of immune response-associated proteins (part of the brain-body stress system), in concert with certain clinical risk factors for FS, may distinguish epileptic from FS episodes with a sensitivity of over 80% and a specificity of over 90% [29]. Whether children show a similar pattern of findings is a subject for future research.

## Approaches to the Treatment of FND

Over the past decade, various treatment approaches for children with FND have been described in the literature, including Retraining and Control Therapy (ReACT), other cognitive-behavioral therapies, multidisciplinary rehabilitation, and treatment as usual (Table 1).

**Table 1:** Treatment studies in pediatric FND over the past decade

**Study**	***n***	**Description**	**Key points**
**Randomized controlled treatment studies**			
Fobian et al. [30•]	29	Randomized, controlled trial of Retraining and Control Therapy (ReACT) vs. supportive therapy control *Duration and setting*: 8 sessions of outpatient ReACT or supportive therapy *Outcome measures*: number of functional seizures; resolution of functional seizures; anxiety; depression	All children had functional seizures; 10% had comorbid epilepsy 52% had clinically significant scores for anxiety, depression, or both Children in ReACT had significantly improved frequency of functional seizures at 7 days posttreatment compared to supportive therapy, with 100% of patients experiencing no functional seizures in the 7 days after ReACT; additionally, 82% remained free of functional seizures for 60 days after ReACT Significant improvements in functional seizures occurred after ReACT, independently of changes in anxiety or depression
**Multidisciplinary rehabilitation studies (prospective)**			
Butz et al. [37]	100	Prospective cohort study of pediatric multidisciplinary rehabilitation *Duration and setting*: 10.5 days (mean; range, 2 − 103 days), inpatient *Outcome measure*: WeeFIM	All children had motor FND; 94/100 (94%) completed the program Treatment included physiotherapy, occupational therapy, recreational therapy, schooling support, and psychotherapy 85% of children reached the maximum WeeFIM score at discharge (full recovery sustained at 2 months) Return to school rates were not reported Comorbid mental health conditions were not reported
Kozlowska et al. [38]	57 60 25	Three prospective cohort studies of multidisciplinary rehabilitation *Duration and setting*: 1 − 3 weeks, inpatient *Outcome measures*: GAF, resolution of FND, return to school, comorbid DSM-5 diagnoses *Comorbid mental health conditions and outcomes* *Factors associated with outcome*: early diagnosis	Children with mixed FND (cohort 1), functional seizures ± other FND symptoms (cohort 2), and mixed FND (cohort 3) Treatment included physiotherapy, psychotherapy (individual and family), attendance at hospital school, and reintegration to home school post discharge FND symptoms resolved in 54/57 (95%), 51/60 (85%), and 22/25 (88%), respectively 45/57 (78.9%), 39/60 (65%), and 14/25 (56%), respectively, returned to full-time school On presentation 41/57 (72%), 38/60 (69%), and 20/25 (80%), respectively, had mental health disorders (mostly anxiety and depression) Children whose existing mental health disorders did not resolve and children who developed chronic mental health disorders later (after their FND had resolved)—11/57 (19%), 22/60 (37%), and 10/25 (40%), respectively—had poorer global functional outcomes Early diagnosis of functional seizures (< 3 months from onset) in cohort 2 was associated with better outcomes [34•]
**Multidisciplinary rehabilitation studies (retrospective)**			
Kozlowska et al. [35]	56	Retrospective cohort study of multidisciplinary rehabilitation *Duration and setting*: 2 − 3 weeks, inpatient *Outcome measures*: resolution of FND, return to school *Comorbid mental health conditions*	Children with mixed FND (± pain) Treatment included physiotherapy, psychotherapy (individual and family), attendance at hospital school, and reintegration to home school post discharge FND symptoms resolved in 35/56 (63%), relapsed temporarily with stress in 10/56 (18%), became chronic in 7/56 (13%), and were unknown in 4/56 (7%) 47/56 (84%) returned to school; one transferred to distance education; one dropped out of school; and data were missing for 4 Anxiety was present in 27/56 (48%), depression in 8/56 (14%), and mixed anxiety and depression in 8/56 (14%) Outcomes for comorbid mental health conditions were not reported
Bolger et al. [39]	30	Retrospective cohort study of pediatric multidisciplinary rehabilitation *Duration and setting*: 8.4 ± 4.2 days, inpatient *Outcome measures*: WeeFIM, return to school	25/30 (83%) children had motor FND as part of their clinical presentations Treatment included physiotherapy, occupational, recreational, and music therapy, and psychological support WeeFIM score change of 30 ± 11.9 (p < .001), maintained at 3 months 20/30 (66.6%) of children had returned to school at 3 months (2 had subsequent psychiatric admissions precluding return to school, and data were missing for 5) Comorbid mental health conditions were not reported
**Cognitive-behavioral therapy studies (+ multimodal multidisciplinary interventions as needed)**			
Sawchuk and Buchhalter [40]	29	Retrospective cohort study *Duration and setting*: hospital-based neurology/psychology service (consecutive referrals over 6 years) *Outcome measures*: full or partial remission (≥ 50% reduction in events) *Comorbid mental health conditions and concerns* *Factors associated with outcome*: acceptance of diagnosis	Children with functional seizures 27/29 (93%) had outpatient psychological treatment that included education around diagnosis (all patients) and CBT (25/29 [86%]), ± psychiatric medication or family therapy; length of treatment ranged 1 − 12 months 17/29 (59%) had full remission, and 6/29 (21%) had partial remission, of their functional seizures on discharge from service 15/29 (52%) had comorbid depression, 6/29 (21%) had comorbid anxiety, and 11/29 (38%) had attention, speech, or learning disorders; 17/20 (85%) evidenced maladaptive personality patterns consistent with passive/avoidant coping strategies Acceptance of the diagnosis at point of assessment by the psychological service
Sawchuk et al. [36•]	43	Retrospective cohort study *Duration and setting*: hospital-based neurology/psychology service (consecutive referrals over 5 years) *Outcome measures*: full or partial remission (≥ 50% reduction in events) *Comorbid mental health conditions* *Factors associated with outcome*: early diagnosis	Children with functional seizures Psychological treatment was stepped: *Level 1*: education regarding diagnosis and management recommendations (all patients); *Level 2*: standardized CBT and biofeedback (16/43 [37%]); *Level 3*: multidisciplinary outpatient intervention (22/43 [51%]); *Level 4*: multidisciplinary inpatient intervention (2/43 [5%] Length of treatment ranged from 1 − 24 months 17/43 (59%) had full remission; 6/43 (21%) had partial remission; and 2/43 had a chronic course > 50% had comorbid mental health disorders, with anxiety, depression, learning difficulties, and self-harm/suicidality being the most common Time to diagnosis > 12 months was associated with lower remission rates
**Treatment as usual (unspecified)**			
Ani et al. [8]	204	Epidemiology study via British Paediatric Surveillance Unit *Duration and setting*: 161/204 (79%) children were treated as inpatients, and 147/204 (72%) had data at 1-year follow-up *Outcome measures*: improvement, no improvement, or worsening of symptoms *Comorbid mental health conditions and outcomes*	Children with mixed FND Treatment via inpatient admission (interventions generally involved a multidisciplinary team) for 161/204 (79%) children At 1-year follow-up, data for 240/469 (51%) symptoms were available; most FND symptoms 217/240 (90%) had improved, 17/240 (7%) had not improved, and 6/240 (3%) were worse; an overall FND remission rate of > 75% was given On presentation, 44/204 (22%) children had mental health disorders (mostly anxiety and depression) On follow-up, 32/115 (28%) children with completed data had been diagnosed with new psychiatric disorders (mostly anxiety and depression) during the follow-up period
Yadav et al. [41]	90	Retrospective cohort study *Duration and setting*: 2-year outpatient follow-up with neurology and psychiatry (treatment unspecified) *Outcome measure:* resolution of FND *Comorbid mental health conditions* *Factors associated with outcome*: early diagnosis, early remission post diagnosis, comorbid diagnosis of epilepsy	Children with functional seizures Treatment was not specified At 2-year follow-up, functional seizures had completely resolved in 32/90 (36%), were generally resolved but with some relapse in 28/90 (31%), and were chronic in 30/90 (33%) On presentation, 60/90 (67%) had mental health disorders (mostly anxiety and depression) Outcomes for comorbid mental health conditions were not reported Early diagnosis (before symptoms were chronic) and early remission were associated with resolution of functional seizures; late diagnosis (when symptoms were becoming chronic) and comorbid diagnosis of epilepsy were associated with chronic functional seizures
Raper et al. [42]	124	Retrospective cohort study *Duration and setting*: 8-year (median) follow-up at transition to adult medical services *Outcome measure*: diagnosis of FND at transition to adult medical services (FND relapse) *Comorbid mental health conditions* *Factors associated with outcome*	Children with mixed FND; 114 reached age 16 years by study census date and transitioned to adult medical services On entrance to adult medical services, 26/114 (23%) sought treatment for FND (relapsing FND); 18/26 (69%) presented with relapses of the same symptom(s) exhibited in their childhood; and 8/26 (31%) presented with different functional neurological symptoms 33/122 (27%) had mental health disorders on presentation to the pediatric service (anxiety and learning disability being the most common) Outcomes for comorbid mental health conditions on transition to adult services were not reported No factors that associated with FND relapse were identified

*CBT* cognitive-behavioral therapy, *WeeFIM* Functional Independence Measure for Children

With the exception of treatment as usual, which varies widely from one institution or provider to another, these approaches are largely biopsychosocial in character [43, 44] (see Text Box 1). As such, they are central to the holistic treatment process required to help children who present with FND [6, 45]. Under these biopsychosocial approaches, treating clinicians consider the biological, psychological, relational, and school-related factors—and the interactions between them—that contribute to the child’s clinical presentation and that may need to be addressed in treatment (see Text Box 2).

Text Box 1 Key Elements of the Biopsychosocial Approach in Working with Children with FND In working with children with FND, the biopsychosocial approach includes the following key elements:

- A comprehensive assessment with the child and family
- In cooperation with the child and family, co-constructing a formulation, which is a summary of the physical, psychological, and social dimensions of the presentation (different system levels) [46, 47]
- The development of a treatment plan (on one or more system levels), as guided by the formulation

Text Box 2 System and Subsystem Levels in the Treatment of FND in Children

**Table:** 

**Biological system level**
Neurology assessment (including comprehensive medical workup)
Neurophysiological regulation (bottom-up interventions) [48, 49]
Physical therapy [37, 50, 51]
Occupational therapy[31, 52]
Speech therapy [31, 53]
Movement retraining via habit reversal for episodic symptoms [30•]
Use of movement and rhythm as neurophysiological and emotional regulation strategies [32, 50, 51]
**Psychological/cognitive system level**
Behavioral interventions that target particular areas via, for example, sleep routines, time scheduling, increasing engagement in enjoyable activities, or decreasing maladaptive behaviors used to avoid or prevent symptoms (sometimes called *safety behaviors*)^*^
Cognitive approaches that target catastrophic symptom expectations and other maladaptive cognitions, thinking patterns, and psychological processes [30•, 31]
Learning interventions for children with identified learning difficulties
Emotion-regulation interventions [32, 33]
**Family system level**
Biopsychosocial assessment with the child and family
Co-construction of a formulation with the child and family
Psychoeducation provided to family regarding FND diagnosis and its predisposing, precipitating, and perpetuating factors [30•, 34•, 35, 36•]
Redirecting the focus of attention of all family members away from FND symptoms
Family interventions to enable the family to support the child’s treatment: decreasing family accommodations to the illness, encouraging the child to use regulation strategies/habit-reversal skills, and other strategies independently, and using motivators to reinforce functional skills and adaptive skills, and to minimize the sick role [30•, 34•, 35]
Other formal family therapy interventions to address family conflict, marital conflict, unresolved grief issues, or issues pertaining to maltreatment
**Social system level**
Reintegration into social life (e.g., time with friends, sports, dance, band)
Attendance/reintegration at the child’s school, which may require a broad range of school-based interventions and collaboration with the school
Development of a brief social script to respond if peers ask about symptoms
Interventions with youth group leaders
Interventions pertaining to social media abuse (with child protection services or police)
Child protection interventions (with child protection services)

^*^An example of maladaptive behavior that is used to avoid symptoms (= safety behaviors) include a child’s having to leave school early and take a nap if he or she child feels strange, in order to prevent a functional seizure

### Retraining and Control Therapy for Functional Seizures

In 2020, Fobian and colleagues published the first and only randomized, controlled trial (RCT) for any pediatric FND treatment [30•]. The study evaluated the efficacy of ReACT, a short-term, outpatient, cognitive-behavioral therapy (CBT)-based intervention for functional seizures (FS), versus supportive therapy, in 29 randomized participants (ReACT *n* = 17; supportive therapy *n* = 12). After an average of 4.6 ReACT sessions, all children in the ReACT group had complete resolution of FS episodes at 7 days posttreatment, with 82% remaining FS-free at 60 days posttreatment. These outcomes were significantly better than the supportive therapy group, which had no significant improvement in FS in the 7 days posttreatment.

ReACT aims to target sense of control and catastrophic symptom expectations and is the first treatment for FND to use principles of habit reversal to retrain physical symptoms. ReACT includes four main components: (1) psychoeducation based on the integrated etiological summary model [54], (2) an individually tailored patient plan to retrain FS symptoms by increasing the child’s sense of control through the use of behaviors incompatible with FS and by challenging catastrophic symptom expectations, (3) a family plan for responding to FS episodes by monitoring for safety while otherwise minimally attending to the child and not interfering with the child’s retraining plan (in [2] above), and (4) a plan to return to school and other social activities.

### Cognitive-Behavioral Therapy as Part of a Pediatric, Stepped-Care Pathway

General CBT has been evaluated as part of a pediatric, stepped-care pathway for children with FS in two recent studies [36•, 40]. The efficacy of CBT in and of itself was not specifically assessed since it was a component of a broader, stepped-care multimodal treatment program. The intervention included one or more of the following: education regarding the diagnosis, bottom-up regulation interventions (e.g., slow-breathing biofeedback), CBT (including trauma-focused CBT when needed), psychiatric medication for comorbid anxiety or depression, intervention for learning difficulties, family therapy, and (for a small subset) inpatient admission. At discharge from the treatment program, 59 − 63% of the children had full remission, and 21 − 28% had partial remission. These outcomes suggest that a traditional CBT approach may be helpful when combined with other interventions.

Although CBT is often thought of as a single treatment, the term *CBT* includes a wide range of techniques that vary by the individual patient, the disorder being treated, and the specific mechanisms being targeted (see Text Box 3) [55]. Moreover, the “dominant assumptions, methods, and goals” of CBT have also changed over time [56, 57, 58]. Consequently, in the absence of an established manualized intervention, the procedures and techniques used in various CBT treatments likely also vary by the individual therapist and the target of the treatment (see Text Box 3). These differences likely affect the outcomes of the different interventions. Additional research is needed to determine the most effective treatment targets, identify which CBT components best effect change on those targets, and develop clear guidelines for using CBT for treating FND [59].

Text Box 3 Cognitive-Behavioral Therapy: The Three Waves

**Table:** 

**Wave 1: Behavior therapy**
In the first wave, behavior therapy methods focus on changing overt behavior by observing, predicting, and modifying behavior to promote health and well-being. Behavior therapy involves learning through association and utilizing reinforcement and punishment to modify behaviors. This wave is based on the work of Ivan Pavlov, Burrhus Frederic Skinner, and John Watson.
**Wave 2: Classic CBT**
The second wave of CBT—based on the work of Albert Ellis and Aaron Beck—focuses on the top-down link between maladaptive cognitions and behaviors; the goal is to detect and alter these existing maladaptive patterns and to develop more adaptive ones by identifying, labeling, and reframing cognitive distortions. This wave of CBT also acknowledges the role of behavior in reinforcing cognitions and feelings and incorporates bottom-up techniques such as exposure and habit reversal.
**Wave 3: Acceptance CBT**
The third wave of CBT is focused on the person’s relationship to thought and emotion more than the content itself. It emphasizes mindfulness (beginning with the work of Jon Kabat-Zinn), emotions, acceptance, values, and meta-cognition. This wave involves top-down, mindfulness-based, and emotion-regulation strategies in which the child utilizes intentional efforts to increase attention and awareness capacities for better control of thoughts and feelings. The objective in third-wave CBT is to help the individual learn to live with painful or unpleasant sensations and with pain in the world and to accept how things are instead of suffering by trying to change them.
**CBT for FND**
Each of the CBT-based interventions for FND utilizes different techniques selected from the above three waves. For example, ReACT uses bottom-up strategies, such as principles of habit reversal and mindfulness, to develop opposing responses to FS symptoms, and it challenges catastrophic symptoms expectations [30•]. Children are asked to attend to their immediate experience (e.g., what they see and hear and their physical sensations) immediately prior to the onset of an FS episode, and then to remain aware and conscious of their current experience while engaging in their opposing responses to prevent or stop FS symptoms. Other interventions [34•, 36•] use bottom-up regulation strategies (e.g., slow-breathing techniques, heart rate variability biofeedback, and grounding techniques [similar to those described for ReACT]) [48] to increase capacity for neuroregulation before implementing other CBT strategies (to target specific symptoms or to target maladaptive cognitions and behaviors).

© Kasia Kozlowska, Areti Vassilopoulos, & Aaron D. Fobian 2021

*CBT* cognitive-behavioral therapy, *FND* functional neurological disorder, *FS* functional seizures

### Multidisciplinary Rehabilitation

To date, both prospective [34•, 37, 38] and retrospective [35, 39] studies have examined multidisciplinary inpatient rehabilitation treatment for FND. Studies have included FND as a broad category (mixed FND, including all presentations and comorbidities) and also specific symptom presentations (e.g., functional gait disorder, FS (with comorbidities)) [34•, 35, 37–39] (see Table 1). Outcomes were very good, with 63 − 95% of children attaining full remission of FND symptoms (see Table 1).

Each multidisciplinary rehabilitation program involved the same key elements in the following domains: focus of treatment, multidisciplinary team/interventions, and post-discharge planning. (1) *Focus of treatment*. All programs implemented a variety of interventions—physical, psychological, family, and school—to facilitate return to normal function. Function was assessed via the Functional Independence Measure for Children (WeeFIM), Global Assessment of Functioning (GAF), participation in school activities, or decreased frequency of health care utilization (e.g., hospital admissions). (2) *Multidisciplinary team/interventions*. All programs used, as needed, physical therapy/occupational therapy, psychotherapy with the child, family therapy, recreational activities, and schoolwork or attendance at the hospital school. In some programs, the role of physiotherapy went beyond restoration of motor function: formalized exercise programs were used to build physical resilience, autonomic regulation, and stress resistance and to contribute to subjective well-being [50, 51]. Psychotherapy with the child included a broad range of approaches. Bottom-up approaches [48, 49, 60] were used to help the child regulate the body’s neurophysiological state [34•]. Top-down approaches such as CBT or talking therapy were used to help with maladaptive thoughts or to work through unresolved grief or other interpersonal issues, respectively [48, 49, 60]. If required, trauma-specific interventions such as Eye Movement Desensitization and Reprocessing (EMDR), radical exposure tapping, or trauma-focused CBT were also used [48, 49, 60]. (3) *Post-discharge planning*. Every inpatient program developed a home discharge plan with the families involving the continuation of step-down supports (e.g., outpatient therapy, follow-up) and school reintegration planning in order to consolidate and maintain functional gains.

Importantly, because most multidisciplinary rehabilitation programs treated children with a broad mix of symptoms and presentations—the programs accepted all children disabled by FND whatever their presentations—clinicians working in these programs put particular emphasis on the role of the biopsychosocial assessment and formulation to understand the particular situation of each child and to guide the treatment process [34•, 35, 37].

### Treatment as Usual (Unspecified)

Some treatment outcomes for treatment as usual or otherwise unspecified interventions are available as part of outcome studies that involve longitudinal follow-up in pediatric FND [8, 41, 42]. A US follow-up study of children with FS found that 36% of children had remission within 6 months (sustained at 2 years) and that 33% never attained remission [41]. No information about treatment was available. A UK study of children with mixed FND reported symptom improvement in 90% of children available at 1-year follow-up (51% of the sample) [8]. As part of treatment as usual, 79% of the children in this cohort had been treated through inpatient admissions, and 69% had a child psychiatrist involved in their care. In another UK study, which evaluated long-term outcomes of FND from childhood to adulthood, Raper and colleagues [42] found that 23% of their sample showed evidence of FND in adulthood at a sufficient level to be documented in their medical records [42]. That level of FND symptoms maintained into adulthood highlights the need for the use of the biopsychosocial interventions discussed above.

### Mental Health Outcomes

Table 1 shows that rates of comorbid mental disorders vary substantially from one cohort to another (22 − 80%) [8, 38, 61]. As with FND that does not resolve, chronic comorbid mental health conditions are associated with long-term effects on social adjustment and health and well-being [6]. Of the studies reviewed in this article, only one study (from Australia) reported long-term outcomes [38]. Kozlowska and colleagues found that 10/57 (18%) of children who had presented with mixed FND 4 years earlier suffered from ongoing mental health problems and lower scores on the Global Assessment of Functioning—despite recovery from FND in 9/10 children. A previous Turkish study had likewise shown that over a third (14/40 or 35%) of children with mixed FND met criteria for an anxiety or mood disorder 4 years later, despite good recovery from FND (34/40 or 85%) [62]. These data suggest that, in a subset of patients, follow-up treatment interventions may need to address comorbid mental health issues long after the resolution of FND.

## Discussion

This review presents a decade of progress in the treatment of children and adolescents with FND. During that time, studies from the USA, Canada, and Australia have documented treatment outcomes from three contemporary specialist treatment programs. Of children treated in such programs, 63 − 95% showed full resolution of FND symptoms. The common thread across the programs is the biopsychosocial approach [43, 44], which has guided the development of different treatment programs across countries and clinical contexts. Today, as a consequence, we have a rich diversity of treatment models and programs. While all the models are embedded in the biopsychosocial model, each program prioritizes certain system levels (see Text Box 2)—or combines interventions on different system levels in different ways—and provides interventions that have been developed to target those system levels.

Progress notwithstanding, much remains to be done. In the remainder of the discussion, we explore some of the challenges pertaining to the treatment of children with FND that face clinicians, researchers, patients, and health care settings. We hope that our discussion of the challenges and issues will help continue the momentum of change.

### Strengthening the Evidence-Base for Clinical Practice

Large, well-conducted RCTs provide the most reliable evidence about treatment efficacy. Currently only one pilot RCT, with a sample of 29- and 60-day follow-up, has been published. This trial provides good preliminary evidence supporting the efficacy of ReACT for children with FS in an outpatient setting. Additional well-powered RCTs are needed, however, to confirm the long-term efficacy of ReACT and other the interventions described above and to identify the most effective treatment targets for individual patients.

A challenge for pediatric researchers is the pervasive heterogeneity of FND regarding all the following domains: FND symptoms, symptom combinations, and levels of functional impairment; comorbid functional somatic symptoms (e.g., pain, fatigue, orthostatic intolerance); comorbid anxiety, depression, and other mental health disorders; and finally, predisposing, precipitating, and perpetuating factors. This complexity in patient presentations is common in many medical and mental health disorders, such as depression, addiction, and hypertension [63, 64, 65, 66, 67]. And it is a complexity that demands the development of adaptive interventions—a type of stepped-care approach [63, 64]—in which the treatment is individually designed, with the treatment strategy, setting, or intensity continually adjusted, over time, to optimize treatment response.

The development of adaptive treatments for pediatric FND may provide enhanced treatment outcomes for patients by providing ways of adjusting treatment for patients who have long-standing or severe symptoms, notable functional impairment, poor treatment response, or multiple comorbidities or complexities. Unfortunately, however, the use of multiple interventions makes RCTs difficult to design and conduct and in any event potentially confounds the outcomes. Recently, to address these issues, sequential, multiple-assignment, randomized trials (SMART) have been used to study adaptive interventions by randomizing participants to different orders of interventions based on specific “decision rules” about when to vary a participant’s treatment [68, 69]. The use of SMART designs in the development of FND interventions will allow controlled evaluation of the most effective ways to tailor individual treatment and to determine the most effective combination of interventions. Given that studies in children indicate that prompt diagnosis and treatment are associated with better outcomes [38, 41], using SMART designs in research, where all the treatment options are considered to be potentially effective active interventions, would eliminate the need to randomize participants to treatment as usual or other control conditions that are known to be ineffective. The results of such research may ultimately help to reduce attrition rates in treatment, as patients who are not benefiting from one treatment will be moved in a timely manner to the next step in a determinate sequence of evidence-based treatments. An alternative to SMART is to evaluate the effectiveness of an intervention systematically across different FND subtypes in consecutive or parallel RCTs.

### Developing a Flexibility of Treatment Models in a Variety of Health Care Contexts

**Fig. 1:**
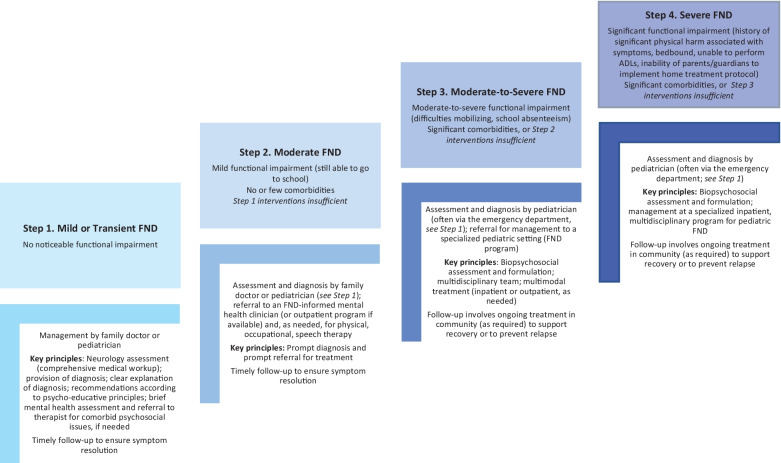
Stepped care approach to functional neurological disorder. Stepped-care model for the management of pediatric functional neurological disorder. For other stepped-care models—developed for functional somatic symptoms more generally—see Schröder and Fink and in Garralda and Rask [44, 70]. © Kasia Kozlowska, Areti Vassilopoulos, & Aaron D. Fobian 2021. ADLs, activities of daily living.

Overall, this review highlights that flexibility is needed in implementing treatment models for pediatric FND. Figure 1 summarizes a stepped-care approach to treatment.

### Limitations

The key limitation of this review is the worldwide dearth of services for FND.^2^ The programs and studies described here reflect the work of individual clinicians—or groups of clinicians—and their local efforts to work with and change service delivery in their health care settings. The dearth of services results, in part, from long-standing stigma, the ingrained belief that patients with FND do not suffer from a real (organic) disorder and that they therefore do not require, or even deserve, treatment [31, 71]. In the wake of recent research advances [1–3], however, far-reaching educational efforts are currently under way to ensure that neurologists and other physicians understand FND as a multi-network brain disorder involving complex interactions between biological, psychological, and social components. Efforts are also under way to promote collaboration between neurologists, mental health clinicians, physical therapists, and other allied health professionals to enable provision of holistic (biopsychosocial) treatment. Nevertheless, compared to other areas of health care, the ongoing shortfall in funding and service delivery for FND—coupled with the time lags associated with research translation—is enormous, leaving patients with FND, worldwide, struggling to obtain adequate treatment.

## Conclusion

Research on pediatric FND treatment provides strong support for current clinical practice. It also offers a foundation for a stepped approach to treatment. Stepped care coupled with a biopsychosocial formulation serves as a framework for an individualized treatment process in pediatric FND. Within the context of available health care resources, stepped care is initially tailored to the needs of the individual child (and family) based on the pattern and severity of FND presentation. The level and type of intervention are then adjusted to take into account the child’s response, over time, to particular treatments or treatment combinations (see Fig. 1). Future research is needed to confirm effective treatment targets, to inform the development of stepped care, and to improve methodologies that can assess the efficacy of stepped-care interventions. More broadly, the health care system needs to improve access to treatments and treatment providers and to undertake further efforts to reduce patient- and provider-related stigma relating to FND and other functional disorders.

## Data Availability

Not applicable.

## Footnotes

1 There are ongoing debates about the most appropriate terminology. Other terms still in common use include the following: psychogenic non-epileptic seizures (DSM-5), dissociative (non-epileptic) seizures (ICD-11), dissociative attacks, and non-epileptic attacks. Terms that have been displaced include pseudoseizures and hysterical convulsions.

2 The dearth of services for FND is also a problem in high-income, Western counties. In the USA, the state of Alabama, for example, has no dedicated, comprehensive inpatient program for functional seizures. Even the city of Boston—with its three major medical schools (Harvard, Tufts, and Boston University)—has no designated outpatient or inpatient services for pediatric FND. Because of the constant flow of referrals, however, Harvard-affiliated Spaulding Rehabilitation Hospital is now working to develop an inpatient FND program. In Australia, the city of Sydney has an inpatient program, but only for the most disabled children, and has no outpatient services. In Canada, no designated or specialized services are available for FND. Clinicians at Alberta Children’s Hospital have developed a clinical care pathway for functional seizure management within the epilepsy clinic [36] but rely on community services for continued follow-up. Clinicians there have also established (in 2018) an outpatient somatic rehabilitation clinic for severe somatic symptom disorders but with limited capacity to address most FND cases.

## References

[1] Perez DL, Nicholson TR, Asadi-Pooya AA, Begue I, Butler M, Carson AJ, et al. Neuroimaging in functional neurological disorder: state of the field and research agenda. Neuroimage Clin. 2021;30:102623. 10.1016/j.nicl.2021.102623 PMC8111317 34215138

[2] Szaflarski JP, LaFrance WC (2018). Psychogenic nonepileptic seizures (PNES) as a network disorder - evidence from neuroimaging of functional (psychogenic) neurological disorders. Epilepsy Curr.

[3] Radmanesh M, Jalili M, Kozlowska K (2020). Activation of functional brain networks in children and adolescents with psychogenic non-epileptic seizures. Front Hum Neurosci.

[4] Leary PM (2003). Conversion disorder in childhood-diagnosed too late, investigated too much. J R Soc Med.

[5] Operto FF, Coppola G, Mazza R, Pastorino GMG, Campanozzi S, Margari L, et al. Psychogenic nonepileptic seizures in pediatric population: a review. Brain Behav. 2019;9(12):e01406. 10.1002/brb3.1406 PMC6908892 31568694

[6] Asadi-Pooya AA, Brigo F, Kozlowska K, Perez DL, Pretorius C, Sawchuk T, et al. Social aspects of life in patients with functional seizures: closing the gap in the biopsychosocial formulation. Epilepsy Behav. 2021;117:107903. 10.1016/j.yebeh.2021.107903 33740497

[7] Stephen CD, Fung V, Lungu CI, Espay AJ (2021). Assessment of emergency department and inpatient use and costs in adult and pediatric functional neurological disorders. JAMA Neurol.

[8] Ani C, Reading R, Lynn R, Forlee S, Garralda E (2013). Incidence and 12-month outcome of non-transient childhood conversion disorder in the UK and Ireland. Br J Psychiatry.

[9] Kozlowska K, Nunn KP, Rose D, Morris A, Ouvrier RA, Varghese J (2007). Conversion disorder in Australian pediatric practice. J Am Acad Child Adolesc Psychiatry.

[10] Pringsheim T, Ganos C, McGuire JF, Hedderly T, Woods D, Gilbert DL, et al. Rapid onset functional tic-like behaviors in young females during the COVID-19 pandemic. Mov Disord. 2021. 10.1002/mds.28778 PMC8441698 34387394

[11] Han VX, Kozlowska K, Kothur K, et al. Rapid onset functional tic- like behaviours in children and adolescents during COVID-19: clinical features, assessment, and biopsychosocial treatment approach. J Paediatr Child Health. 2022. 10.1111/jpc.15932 PMC9115185 35247213

[12] AmericanPsychiatric A (2013). Diagnostic and statistical manual of mental disorders: DSM-5.

[13] World Health Organization. International statistical classification of diseases and related health problems, 11th Revision (ICD-11). https://icd.who.int/en. Geneva: World Health Organization. 2018. Accessed 1 Oct 2021.

[14] de Gusmao CM, Guerriero RM, Bernson-Leung ME, Pier D, Ibeziako PI, Bujoreanu S (2014). Functional neurological symptom disorders in a pediatric emergency room: diagnostic accuracy, features, and outcome. Pediatr Neurol.

[15] Stone J (2014). Functional neurological disorders: the neurological assessment as treatment. Neurophysiol Clin.

[16] Carson A, Lehn A, Ludwig L, Stone J (2015). Explaining functional disorders in the neurology clinic: a photo story. Pract Neurol.

[17] Kozlowska K, Mohammad S. Functional neurological disorder in children and adolescents: assessment and Treatment. In: Sivaswamy L, Kamat D, editors. Symptom Based Approach to Pediatric Neurology: Springer Nature; forthcoming in 2022.

[18] Espay AJ, Aybek S, Carson A, Edwards MJ, Goldstein LH, Hallett M (2018). Current concepts in diagnosis and treatment of functional neurological disorders. JAMA Neurol.

[19] Heyman I, Liang H, Hedderly T. COVID-19 related increase in childhood tics and tic-like attacks. Arch Dis Child. 2021. 10.1136/archdischild-2021-321748 33677431

[20] Ibrahim NM, Martino D, van de Warrenburg BP, Quinn NP, Bhatia KP, Brown RJ (2009). The prognosis of fixed dystonia: a follow-up study. Parkinsonism Relat Disord.

[21] Thomas M, Vuong KD, Jankovic J (2006). Long-term prognosis of patients with psychogenic movement disorders. Parkinsonism Relat Disord.

[22] Khachane Y, Kozlowska K, Savage B (2019). Twisted in Pain: The Multidisciplinary Treatment Approach to Functional Dystonia. Harv Rev Psychiatry.

[23] De Paola L, Terra VC, Silvado CE, Teive HA, Palmini A, Valente KD (2016). Improving first responders’ psychogenic nonepileptic seizures diagnosis accuracy: development and validation of a 6-item bedside diagnostic tool. Epilepsy Behav.

[24] LaFrance WC, Baker GA, Duncan R, Goldstein LH, Reuber M (2013). Minimum requirements for the diagnosis of psychogenic nonepileptic seizures: a staged approach: a report from the International League Against Epilepsy Nonepileptic Seizures Task Force. Epilepsia.

[25] Xiang X, Fang J, Guo Y. Differential diagnosis between epileptic seizures and psychogenic nonepileptic seizures based on semiology. Acta Epileptologica. 2019;1(6).

[26] Leibetseder A, Eisermann M, LaFrance Jr WC, Nobili L, von Oertzen TJ. How to distinguish seizures from non-epileptic manifestations. Epileptic Disord. 2020;22(6):716-738 10.1684/epd.2020.1234 33399092

[27] Braun M, Sawchuk T, Simpkins A, Heer N, Johnson J, Esser M, et al. Quantitative EEG during hyperventilation as a biomarker for pediatric psychogenic non-epileptic seizures (PNES). Poster session presented at the annual meeting of the American Epilepsy Society, Chicago, USA. 2021.

[28] Kozlowska K, Rampersad R, Cruz C, Shah U, Chudleigh C, Soe S (2017). The respiratory control of carbon dioxide in children and adolescents referred for treatment of psychogenic non-epileptic seizures. Eur Child Adolesc Psychiatry.

[29] Gledhill JM, Brand EJ, Pollard JR, St Clair RD, Wallach TM, Crino PB (2021). Association of epileptic and nonepileptic seizures and changes in circulating plasma proteins linked to neuroinflammation. Neurology.

[30] Fobian AD, Long DM, Szaflarski JP (2020). Retraining and control therapy for pediatric psychogenic non-epileptic seizures. Ann Clin Transl Neurol.

[31] Kozlowska K, Sawchuk T, Waugh JL, Helgeland H, Baker J, Scher S, et al. Changing the culture of care for children and adolescents with functional neurological disorder. Epilepsy and Behavior Reports. 2021;16 (1004486). 10.1016/j.ebr.2021.100486 PMC8567196 34761194

[32] Shafir T. Using movement to regulate emotion: neurophysiological findings and their application in psychotherapy. 2016;7(1451). 10.3389/fpsyg.2016.01451 PMC5033979 27721801

[33] Castagna PJ, Shelley R. Upton SR, Long ACJ. Cognitive behavior therapy for children with emotion regulation challenges In: Maykel C, Bray MA, editors. Promoting Mind–Body Health in Schools: Interventions for Mental Health Professionals. Washington DC: American Psychological Association. 2019; 317–33.

[34] Kozlowska K, Chudleigh C, Cruz C, Lim M, McClure G, Savage B (2018). Psychogenic non-epileptic seizures in children and adolescents: Part II - explanations to families, treatment, and group outcomes. Clin Child Psychol Psychiatry.

[35] Kozlowska K, English M, Savage B, Chudleigh C, Davies F, Paull M, et al. Multimodal rehabilitation: a mind-body, family-based intervention for children and adolescents impaired by medically unexplained symptoms. Part 2: Case studies and outcomes. Am J Fam Ther. 2013;41(3):212–31.

[36] • Sawchuk T, Buchhalter J, Senft B. Psychogenic nonepileptic seizures in children-prospective validation of a clinical care pathway & risk factors for treatment outcome. Epilepsy Behav. 2020;105:106971. This prospective study demonstrates that standardized care (including bottom-up regulation strategies and cognitive behavioral therapy) for pediatric functional seizures is associated with improved clinical outcomes and reduced healthcare utilization. 10.1016/j.yebeh.2020.106971 32126506

[37] Butz C, Iske C, Truba N, Trott K (2019). Treatment of functional gait abnormality in a rehabilitation setting: emphasizing the physical interventions for treating the whole child. Innovations in clinical neuroscience (ICNS).

[38] Kozlowska K, Gray N, Scher S, Savage B (2020). Psychologically informed physiotherapy as part of a multidisciplinary rehabilitation program for children and adolescents with functional neurological disorder: physical and mental health outcomes. J Paediatr Child Health..

[39] Bolger A, Collins A, Michels M, Pruitt D (2018). Characteristics and outcomes of children with conversion disorder admitted to a single inpatient rehabilitation unit, a retrospective study. PM R.

[40] Sawchuk T, Buchhalter J (2015). Psychogenic nonepileptic seizures in children - psychological presentation, treatment, and short-term outcomes. Epilepsy Behav.

[41] Yadav A, Agarwal R, Park J (2015). Outcome of psychogenic nonepileptic seizures (PNES) in children: a 2-year follow-up study. Epilepsy Behav.

[42] Raper J, Currigan V, Fothergill S, Stone J, Forsyth RJ. Long-term outcomes of functional neurological disorder in children. Arch Dis Child. 2019;104(12):1155–60. 10.1002/9781118381953 https://onlinelibrary-wiley-com.ezp-prod1.hul.harvard.edu/doi/pdf/10.1002/9781118381953.ch72. Accessed 1 Oct 2021. 31326916

[43] Engel GL (1980). The clinical application of the biopsychosocial model. Am J Psychiatry.

[44] Garralda ME, Rask CU. Somatoform and related disorders. In: Thapar A, Pine DS, Leckman JF, Scott S, Snowling MJ, Taylor E, editors. Rutter’s child and adolescent psychiatry. Sixth edition ed. Chichester, West Sussex ; Ames, Iowa : Wiley: John Wiley & Sons. 2015;1035–54. 10.1002/9781118381953https://onlinelibrary-wiley-com.ezp-prod1.hul.harvard.edu. Accessed 1 Oct 2021.

[45] Perez DL, Edwards MJ, Nielsen G, Kozlowska K, Hallett M, LaFrance WC (2021). Decade of progress in motor functional neurological disorder: continuing the momentum. J Neurol Neurosurg Psychiatry.

[46] Henderson S, Martin A. Case formulation and integration of information in child and adolescent mental health. In: Rey JM, editor. IACAPAP e-textbook of child and adolescent mental health. Geneva: Int Assoc Child Adolesc Psychiatry Allied Prof. 2014. https://iacapap.org/content/uploads/A.10-CASE-FORMULATION-2014.pdf. Accessed 1 Oct 2021.

[47] Winters NC, Hanson G, Stoyanova V (2007). The case formulation in child and adolescent psychiatry. Child Adolesc Psychiatr Clin N Am..

[48] Kozlowska K, Scher S, Helgeland H (2020). Functional somatic symptoms in children and adolescents: the stress-system approach to assessment and treatment.

[49] Velani H, Gledhill J (2021). The effectiveness of psychological interventions for children and adolescents with non-epileptic seizures. Seizure..

[50] Gray N, Savage B, Scher S, Kozlowska K (2020). Psychologically informed physical therapy for children and adolescents with functional neurological symptoms: the wellness approach. J Neuropsychiatry Clin Neurosci.

[51] Kim Y, Gray N, Jones A, Scher S, Kozlowska K. The role of physiotherapy in the management of functional neurological disorder in children and adolescents. Semin Pediatr Neurol 2021. 10.1016/j.spen.2021.100947 35450664

[52] Weiss KE, Steinman KJ, Kodish I, Sim L, Yurs S, Steggall C (2021). Functional neurological symptom disorder in children and adolescents within medical settings. J Clin Psychol Med Settings.

[53] Baker J, Barnett C, Cavalli L, Dietrich M, Dixon L, Duffy JR (2021). Management of functional communication, swallowing, cough and related disorders: consensus recommendations for speech and language therapy. J Neurol Neurosurg Psychiatry..

[54] Fobian AD, Elliott L. A review of functional neurological symptom disorder etiology and the integrated etiological summary model. J Psychiatry Neurosci. 2018;43(5):170190. 10.1503/jpn.170190 PMC6306282 30565902

[55] Beck AT (1976). Cognitive therapy and the emotional disorders.

[56] Hayes SC (2016). Acceptance and commitment therapy, relational frame theory, and the third wave of behavioral and cognitive therapies - Republished Article. Behav Ther.

[57] Brown LA, Gaudiano BA, Miller IW (2011). Investigating the similarities and differences between practitioners of second- and third-wave cognitive-behavioral therapies. Behav Modif.

[58] Kabat-Zinn J (2003). Mindfulness-based interventions in context: past, present, and future. Clin Psychol Sci Pract.

[59] Fobian AD, Szaflarski JP. Identifying and evaluating novel treatment targets for the development of evidence-based interventions for functional neurological disorder. Epilepsy Behav Rep. 2021;16:100479. 10.1016/j.ebr.2021.100479 PMC8449163 34568805

[60] Cristea IA, Vecchi T, Cuijpers P (2021). Top-down and bottom-up pathways to developing psychological interventions. JAMA Psychiat.

[61] Hansen AS, Rask CU, Christensen AE, Rodrigo-Domingo M, Christensen J, Nielsen RE (2021). Psychiatric disorders in children and adolescents with psychogenic nonepileptic seizures. Neurology.

[62] Pehlivanturk B, Unal F (2002). Conversion disorder in children and adolescents: a 4-year follow-up study. J Psychosom Res.

[63] Glasgow MS, Engel BT, D'Lugoff BC (1989). A controlled study of a standardized behavioral stepped treatment for hypertension. Psychosom Med.

[64] Breslin FC, Sobell MB, Sobell LC, Cunningham JA, Sdao-Jarvie K, Borsoi D (1999). Problem drinkers: evaluation of a stepped-care approach. J Subst Abuse.

[65] Lavori PW, Dawson R, Rush AJ (2000). Flexible treatment strategies in chronic disease: clinical and research implications. Biol Psychiatry.

[66] Untzer J, Katon W, Williams JW, Callahan CM, Harpole L, Hunkeler EM (2001). Improving primary care for depression in late life: the design of a multicenter randomized trial. Med Care..

[67] Brooner RK, Kidorf M (2002). Using behavioral reinforcement to improve methadone treatment participation. Science and Practice Perspectives.

[68] Murphy SA (2005). An experimental design for the development of adaptive treatment strategies. Stat Med.

[69] Lei H, Nahum-Shani I, Lynch K, Oslin D, Murphy SA (2012). A “SMART” design for building individualized treatment sequences. Annu Rev Clin Psychol.

[70] Schroder A, Fink P (2011). Functional somatic syndromes and somatoform disorders in special psychosomatic units: organizational aspects and evidence-based treatment. Psychiatr Clin North Am.

[71] Herbert LD, Kim R, Hassan AAO, Wilkinson-Smith A, Waugh JL. When neurologists diagnose functional neurological disorder, why don’t they code for it? CNS Spectrums. 2021;1-30. 10.1017/S1092852921000833 PMC8920954 34521502

